# The Constructing of the Oxide Phase Diagram for Fluoride Adsorption on La-Fe-Al: A Collaborative Study of Density Functional Calculation and Experimentation

**DOI:** 10.3390/nano14070619

**Published:** 2024-04-01

**Authors:** Shaojian Xie, Yao Xiao, Lei Huang, Jiaxin Li, Jia Yan, Qian Li, Meng Li, Hongguo Zhang

**Affiliations:** 1 School of Environmental Science and Engineering, Guangzhou University, Guangzhou Higher Education Mega Center, Guangzhou 510006, China; xsj-xie@foxmail.com (S.X.); xiaoyaogzhu2023@163.com (Y.X.); lijiaxin9668277@163.com (J.L.); jiayan@gzhu.edu.cn (J.Y.); qianli@gzhu.edu.cn (Q.L.); mengli@gzhu.edu.cn (M.L.); 2 Guangzhou University-Linköping University Research Center on Urban Sustainable Development, Guangzhou University, Guangzhou 510006, China

**Keywords:** fluoride, oxides, adsorbent, phase diagram

## Abstract

In recent years, fluoride pollution in water is a problem that has attracted much attention from researchers. The removal of fluoride-containing wastewater by adsorption with metal oxide as an adsorbent is the most common treatment method. Based on this, the effect of the doping ratio of La_2_O_3_, Fe_2_O_3,_ and Al_2_O_3_ on the fluoride-removal performance was discussed by constructing a phase diagram. In this study, the adsorption mechanism of nanocrystalline lanthanum oxide terpolymer was investigated by density functional theory calculation and experiment. The optimal pH condition selected in the experiment was three, and the adsorption kinetics of fluoride ions were more consistent with the quasi-second-order kinetic model. The adsorption thermodynamics was more consistent with the Langmuir model. When the La-Fe-Al ternary composite oxides achieved the optimal adsorption efficiency for fluoride ions, the mass synthesis ratio was Al_2_O_3_:(Fe_2_O_3_:La_2_O_3_ = 1:2) = 1:100, resulting in a fluoride ion removal rate of up to 99.78%. Density functional calculations revealed that the La-Fe-Al ternary composite oxides had three important adsorption sites for La, Fe, and Al. Among them, the adsorption capacity for HF was Fe_2_O_3_ > La_2_O_3_ > Al_2_O_3_, and for F^−^ was La_2_O_3_ > Al_2_O_3_ > Fe_2_O_3_. This provided good guidance for designing adsorbents to remove fluoride.

## 1. Introduction

Water is the source of life in nature and is closely related to the survival and development of human beings [1]. At present, the freshwater resources that can be used by human beings are mainly groundwater and river-running water [2]. Nevertheless, the release of wastewater from metallurgical and semiconductor industries, the natural degradation of fluoride-containing minerals, and the application of pesticides and fertilizers in agricultural production can lead to fluoride contamination in water bodies [3,4,5,6]. According to relevant studies, the water environment of most countries in the Americas, Asia, and Africa has serious fluoride pollution [7,8,9,10], posing a serious threat to human health. The World Health Organization limits the maximum amount of fluoride ions in drinking water to 1.5 mg/L to avoid the harm caused by fluoride in water [11,12].

At present, the mature wastewater treatment processes for removing fluoride mainly include the adsorption method, ion-exchange method, membrane-separation method, chemical-precipitation method, electric flocculation method, and reverse-osmosis method [13,14,15,16,17]. Of these techniques, the chemical precipitation method is the predominant water treatment technology employed in the industry. However, this method often requires an excessive use of precipitants, resulting in general treatment efficacy and the potential for secondary pollution [18,19,20]. Because of its simple operation, low cost, high removal efficiency, and low secondary pollution, the adsorption method is one of the most studied technologies for treating wastewater containing fluoride [21,22]. The selection of an adsorbent is determined by the adsorption method to remove the efficiency of fluoride wastewater. Common adsorbents for dealing with fluoride mainly include bioderived carbon, transition metal oxide, water talc, zeolite, reactive oxygen aluminum, modified alumina, biopolymer, metal double hydroxide, etc. [23,24,25,26,27].

Among the aforementioned adsorbents, conventional adsorbents typically exhibit drawbacks, such as low adsorption efficiency, high economic expenses, and limited chemical stability. Nevertheless, metal oxides have the advantages of high-efficiency adsorption capacity, significant ion selectivity, good acid and alkali resistance, easy regeneration, and low cost. For example, activated alumina has a large specific surface area and a rich aperture structure. It is the most common kind of fluoride-removal adsorbent [28,29]. Relevant studies have shown that Fe is a typical transition metal, widely distributed in environmental minerals. Fe_2_O_3_ was a very promising adsorbent [30]. However, the adsorption of fluoride ions by single Al_2_O_3_ and Fe_2_O_3_ had certain limitations. In addition, the high-valence state of La(III) could also provide an affinity coordination site for fluoride ions. This non-toxic and harmless element was easy to combine with the O element to form La_2_O_3_, which was a very ideal adsorbent [31,32]. Since the adsorption of fluoride by a single metal oxide had limitations, metal oxides such as Al_2_O_3_, Fe_2_O_3,_ and La_2_O_3_ were doped with each other to form composite products, which might be a relatively novel method, and relevant studies have also proven this point. For example, Wang et al. [33] found that the composite material formed by proportionally doping Fe_2_O_3_ and ZrO_2_ could not only enhance the pH range of fluoride-containing wastewater treatment but also significantly improve the adsorption capacity of fluoride. The adsorption capacity could reach 113.38 mg/g. Zhang et al. [34] combined Fe_3_O_4_ with MgO to form Fe_3_O_4_@MgO material, and the results showed that the removal efficiency of the composite adsorbent for fluoride-containing groundwater reached 97.3%. Dong et al. [35] synthesized a new efficient Mg-Fe-Ce ternary composite oxide adsorbent, and the results showed that the adsorbent has a wide optimal pH processing range (4.0–5.5). The maximum adsorption capacity can reach 204 mg/g, and it exhibits good adsorption performance, even at pH = 7. In summary, it was common for metal oxides to dope with each other to improve adsorption properties, but few researchers had investigated the impact of composites for removing fluoride. Therefore, it is necessary to study the composite ratios and construct adsorption phase diagrams of composite materials. Phase diagrams are important tools in the fields of metallurgy and materials science. By constructing phase diagrams, we can identify the appropriate doping ratios for materials and better explain the impact of composite materials on adsorption performance.

In this work, La-Fe-Al ternary composite oxides were synthesized from ferric chloride, aluminum sulfate, and lanthanum oxide by inter-doping and high-temperature calcination. According to the adsorption effect of La-Fe-Al oxides on the fluorine-containing solution, the phase diagram was constructed, and the optimal synthesis ratio of the material was Al_2_O_3_:(Fe_2_O_3_:La_2_O_3_ = 1:2) = 1:100. The removal rate of fluoride ions could reach 99.78%, and the optimal adsorption pH was three. The structure and composition of La-Fe-Al oxides before and after adsorption were analyzed by scanning electron microscopy (SEM), X-ray diffraction (XRD), Fourier transform infrared spectroscopy (FT-IR), thermogravimetric (TG), and X-ray photoelectron spectroscopy (XPS). The adsorption mechanism of La-Fe-Al oxides on fluoride was described by density functional theory. These results showed that La-Fe-Al ternary composite oxides were very ideal fluoride adsorbents, and the successful construction of a phase diagram provided a research idea for the application of a phase diagram in the adsorption field.

## 2. Experiment

### 2.1. Synthesis of La-Fe-Al Oxides

**Figure 1:**
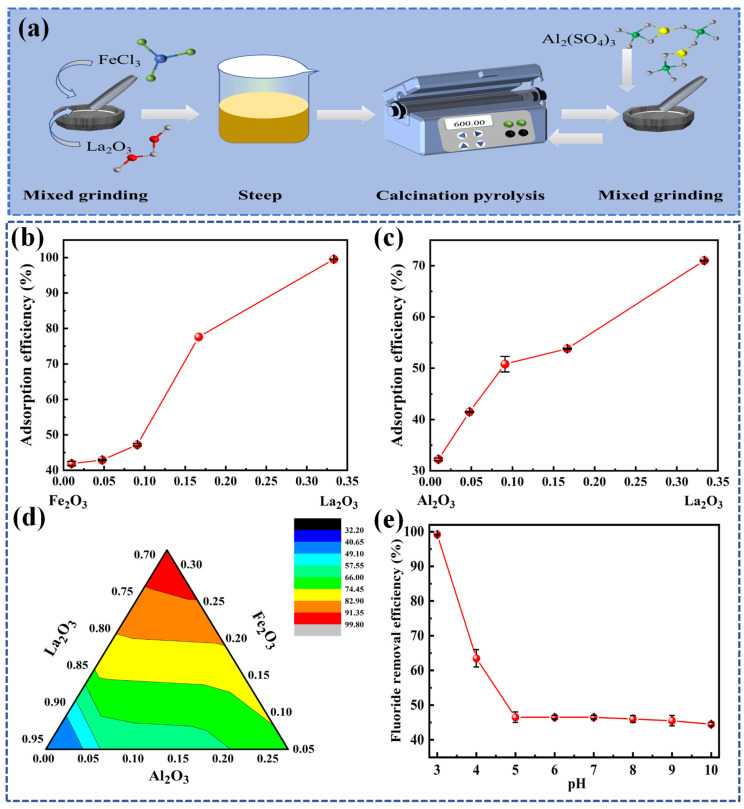
(**a**) Synthesis diagram of La-Fe-Al ternary composite oxides; (**b**) The treatment effect of binary composite products doped with lanthanum oxide and ferric chloride on fluoride ions; (**c**) The treatment effect of binary composite product doped with lanthanum oxide and aluminum sulfate on fluoride ions; (**d**) Phase diagram of La-Fe-Al ternary composite oxides doped with lanthanum oxide, ferric chloride, and aluminum sulfate; and (**e**) Removal efficiency of La-Fe-Al ternary composite oxides for fluoride ions at different pH.

All the experimental reagents were purchased from Shanghai McLean, Tianjin Zhiyuan Chemical, Tianjin Yongda Chemical, and other pharmaceutical companies in China. All the chemical reagents belonged to the analytical pure level (AR). In this experiment, lanthanum oxide was used as the substrate, and aluminum sulfate and ferric chloride were used as dopants, respectively. Two doping methods were designed here. The A method was lanthanum oxide and ferric chloride doping, and the B method was lanthanum oxide and aluminum sulfate doping. The two raw materials were doped according to the mass ratio of 1%, 5%, 10%, 20%, and 50%, and after full grinding, soaked in 20 mL of deionized water for 24 h. Then, they were placed in the oven at 60 °C to dry fully. Finally, after calcination at 600 °C for 24 h (heating rate of 10 °C/min), nano-lanthanum oxide binary composite oxide materials could be obtained. The synthesis of La-Fe-Al ternary composite oxides was based on the binary composite oxides. La-Fe-Al ternary composite oxides could be obtained by mixing aluminum sulfate and ferric chloride with A and B doping methods, respectively, according to the above proportion, and keeping the calcination temperature and time unchanged. Figure 1a shows the synthesis diagram of La-Fe-Al ternary composite oxides.

### 2.2. Physical Characterization Methods

The morphology of the La-Fe-Al ternary oxides was analyzed by a TESCAN MIRA LMS scanning electron microscope (SEM) (Taisken Co., Ltd., Shanghai, China) and a JEOL F200 transmission electron microscope (TEM) (Jieou Road Technology and Trade Co., LTD., Beijing, China). The crystal structure of the La-Fe-Al ternary oxides was analyzed by X-ray diffraction (XRD) (Rigaku Ultima IV) (Nihon Rigaku Kabushiki Kaisha, Tokyo, Japan) to verify whether the oxides were successfully recombined. The elements and chemical-bond composition of the materials were analyzed by the United States Thermo Scientific K-Alpha X-ray photoelectron spectrometer (XPS). The changes of functional groups before and after adsorption were analyzed by Fourier infrared spectroscopy (FTIR) of Thermo Scientific iN10 (Thermo Fisher Scientific Inc., Waltham, MA, USA). The thermal stability of the material was monitored by the TA Discovery TGA 550 thermogravimetric analyzer (TG) (Waters Corporation, Shanghai, China).

### 2.3. Preparation of Fluoride Solution

In the experiment, sodium fluoride solid was prepared with a concentration of 1000 mg/L fluoride ion solution as a diluted mother liquor. Other concentrations of fluoride-containing solutions can be obtained by dilution. First of all, it should be emphasized that the experiment mainly takes the fluoride-containing solution of 100 mg/L as the research object, the treatment volume is 50 mL, and the additional amount of the above-mentioned prepared adsorbent is 50 mg. In the anion competition experiment, the added anions mainly included NO_3_^−^, SO_4_^2−^, Cl^−^, Br^−^, and PO_4_^3−^, and all the concentrations were 10 mmol/L.

### 2.4. Determination of Fluoride Solution Concentration

The masking agent required for the test was prepared with sodium chloride (58 g), sodium citrate (10 g), glacial acetic acid (57 mL), and deionized water (800 mL). After adsorption, 40 mL of fluorine solution and 10 mL of masking agent were mixed to prepare the test samples. Before the test, standard curves were prepared with fluorine ion solutions with concentrations of 1 mg/L, 10 mg/L, 100 mg/L, and 1000 mg/L. A fluoride ion composite electrode was used to test the fluorine concentration. The concentration of fluoride was calculated for the removal efficiency by Equation (1).

(1) $$\eta =\frac{C_{0}-C_{e}}{C_{0}}\times 100\%$$

where η is the removal rate of fluoride ions and C_0_ is the initial concentration of fluoride ions in the solution, expressed in mg/L. C_e_ is the concentration of fluoride ions obtained after adsorption; the unit is mg/L.

### 2.5. Adsorption Model

The adsorption time data are used to simulate the adsorption kinetics. The adsorption kinetics models mainly include the pseudo-first-order kinetic model, pseudo-second-order kinetic model, Elovich kinetic model, internal diffusion kinetic model, and external diffusion kinetic model [36,37].

(2) $$\log\left(q_{e}-q_{t}\right)={\mathrm{logq}}_{e}-\frac{K_{1}t}{2.303}$$

(3) $$\frac{t}{q_{t}}=\frac{1}{K_{2}q_{e}^{2}}+\frac{t}{q_{e}}$$

(4) $$q_{t}=A+2.303\mathrm{Blogt}$$

(5) $$q_{t}=K_{\mathrm{id}}\cdot t^{\frac{1}{2}}+C$$

(6) $$\ln\frac{C_{t}}{C_{0}}=-K_{p}t$$

where t is the adsorption time, q_e_ is the adsorption capacity at equilibrium (mg/g), and q_t_ is the adsorption capacity corresponding to time t (mg/g). K_1_ (min^−1^), K_2_ (g/mg/min), K_id_ (mg/g/min^0.5^), and K_p_ (min^−1^) belong to the relevant parameters of their respective kinetics. A, B, and C are the adsorption kinetic constants, C_t_ is the concentration corresponding to time t (mg/L), and the other relevant parameters are the same as the above equation.

Adsorption data about different concentrations of fluoride were used to simulate the adsorption thermodynamics, which included the Langmuir model, Freundlich model, Temkin model, and Dubinin–Radushkevich model [38,39].

(7) $$Q_{e}=\frac{q_{\max}{\mathrm{bC}}_{e}}{1+{\mathrm{bC}}_{e}}$$

(8) $$q_{e}=K_{F}C_{e}^{1/n}$$

(9) $$q_{e}=\frac{\mathrm{RT}}{b_{T}}\ln\left(K_{T}{\mathrm{TC}}_{e}\right)$$

(10) $$\ln q_{e}=\ln q_{\max}-{\beta \varepsilon }^{2}$$

where q_max_ is the maximum simulated saturated adsorption capacity (mg/g) and b is Langmuir’s constant. In the Freundlich equation, K_F_ (mg^1−(1/n)^ L^1/n^g^−1^) and n are Freundlich constants related to adsorption capacity and adsorption strength. In the Temkin equation, b_T_ and K_T_ are Temkin thermodynamic constants and Temkin equilibrium association constants, respectively. In the Dubinin–Radushkevich formula, ε is the Polanyi adsorption potential correlation constant, and β is the activity coefficient related to the mean adsorption-free energy. The meaning of the other symbols is the same as that of the above formula.

### 2.6. Density Functional Theory Simulation (DFT)

Based on density functional theory, the theoretical simulation calculation uses simulation software to calculate the adsorption energy, generalized gradient approximation (GGA), and basis group PW91 to calculate the change of adsorption energy and the change of adsorption at the atomic level under ultra-high precision conditions. The adsorption of fluoride by the ternary system La-Fe-Al was studied. The adsorption binding energy can be calculated by Equation (11) as follows.

(11) $$E_{\mathrm{AB}}=E_{A-F}-E_{A}-E_{F}$$

where E_AB_ is the adsorption binding energy, E_A–F_ is the energy after binding of the adsorbent, E_A_ is the energy of the adsorbent, E_F_ is the energy of F^−^ or HF, and the unit is kJ/mol.

### 2.7. Operation of the Experiment

Dilute the 1000 mg/L fluoride ion mother liquor into concentrations of 5 mg/L, 10 mg/L, 50 mg/L, 100 mg/L, 200 mg/L, etc. for later use. Take 50 mL of fluoride ion solution with a pipette and add it to a plastic vial; adjust the pH with the HCl solution and NaOH solution. Weigh 50 mg of the prepared composite oxide adsorbent mentioned above, add it to the vial, place it on a shaker, and set the temperature and time. After adsorption is complete, filter the solution and determine the concentration of fluoride ions according to the fluoride ion determination method in Section 2.4.

## 3. Results and Discussion

### 3.1. Adsorption Experiment

#### 3.1.1. Adsorption Phase Diagram

The synthesis diagram of La-Fe-Al ternary composite oxides is illustrated in Figure 1a. The binary phase diagram is shown in Figure 1b,c. It revealed the adsorption effect of the binary composite made by doping lanthanum oxide with different proportions of ferric chloride and aluminum sulfate, respectively. This demonstrated that the most effective mass ratio of nano-lanthanum oxide binary composite oxides for removing fluoride was found to be Fe_2_O_3_:La_2_O_3_ = 1:2, resulting in a fluoride ions removal rate of 99.56%. Based on the best nano-lanthanum oxide binary composite oxides, the ternary composite materials were further investigated. The La-Fe-Al ternary composite oxides were obtained by calcination of aluminum sulfate and ferric chloride mixed with two binary composite products, respectively. The La-Fe-Al ternary composite oxides phase diagram of adsorbing fluoride ions is illustrated in Figure 1d. It could be observed that, when La-Fe-Al ternary composite oxides achieved the best adsorption effect on fluoride ions, the mass ratio of the material was Al_2_O_3_:(Fe_2_O_3_:La_2_O_3_ = 1:2) = 1:100, and the removal rate of fluoride ions could reach 99.78%.

The pH values of 3, 4, 5, 6, 7, 8, 9, and 10 were designed to further explore the effect of pH on the fluoride-removal efficiency of La-Fe-Al ternary composite oxides in Figure 1e. The experimental results indicated that the adsorption efficiency of the adsorbent for removing fluoride ions decreased as the pH increased. When the pH value was greater than five, the removal rate of fluoride ions was scarcely changed with the increase in pH value. Therefore, the optimal pH condition selected in this experiment was three, and the adsorption rate reached 99.78%. In fact, at low pH, the adsorption material is positive, while the fluoride ions in the solution have hydrogen-bond interaction [40]. Therefore, with the decrease in pH, the stronger the ability that HF could dissociate into F^−^. The greater the positive-charge capacity of La-Fe-Al ternary composite oxides, the more effective they are at capturing F^−^ in solution through hydrogen bonding. Thus, they have a higher removal efficiency of fluoride ions.

#### 3.1.2. Adsorption Kinetics

The purpose of the kinetic experiment was to investigate the removal rate of fluoride ions with the increase of adsorption time. First of all, it should be emphasized that the kinetic experiments were carried out at pH = 3. The adsorption time was set as 5 min, 10 min, 30 min, 60 min, 120 min, and 240 min. The fluoride ions’ adsorption kinetics curve of La-Fe-Al ternary composite oxides is illustrated in Figure S1. The adsorption rate of fluoride ions by adsorbed materials was very fast. When the adsorption time was 5 min, the removal rate of fluoride ions reached 74.7%. Within 5 min to 60 min, the adsorption capacity still increased significantly, from 74.7% to 98.6%. After 60 min, the removal rate did not change significantly, indicating that the adsorption equilibrium was reached within 60 min. The adsorption might be mainly achieved through the effective contact between the pore size of the adsorbent and the fluoride ions in the solution within 60 min. With the extension of time, and the more effective contact, the removal rate of fluoride ions was higher.

**Figure 2:**
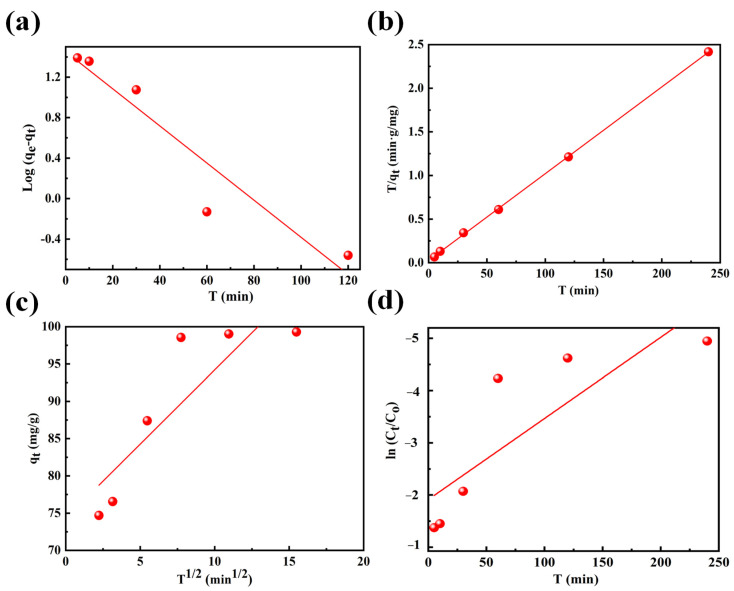
Data fitting of fluoride ion adsorption by La-Fe-Al ternary composite oxides: (**a**) pseudo-first-order kinetic model; (**b**) pseudo-second-order kinetic model; (**c**) intra-particle diffusion kinetic model; and (**d**) external diffusion kinetic model.

In this study, various kinetic models, including the pseudo-first-order, pseudo-second-order, internal diffusion, external diffusion, and Elovich models, were employed to characterize the adsorption process. The fitting results of four models are shown in Figure 2a–d. The fitting results of Elovich’s model can be observed in Figure S2. And the dynamics parameters obtained through fitting are illustrated in Table S1. According to the fitting results, the adsorption kinetic of fluoride ions by ternary composite oxides was more consistent with the pseudo-second-order kinetic model, and the phase-relation value was R^2^ = 0.999. It was higher than that of the pseudo-first-order kinetic model (R^2^ = 0.876), the internal diffusion kinetic model (R^2^ = 0.700), the external diffusion kinetic model (R^2^ = 0.639), and the Elovich model (R^2^ = 0.891). This indicated that the removal of fluoride ions by the composite was mainly based on chemisorption [41,42]. The metal bonds of the La-Fe-Al ternary composite oxides interact with F^−^ ions, forming structures like La-F. In addition, the adsorption capacity calculated by the pseudo-second-order kinetic model (q_e_ = 100.91 mg/g) was closer to the experimental value (q_e_ = 99.69 mg/g).

#### 3.1.3. Adsorption Thermodynamics

**Figure 3:**
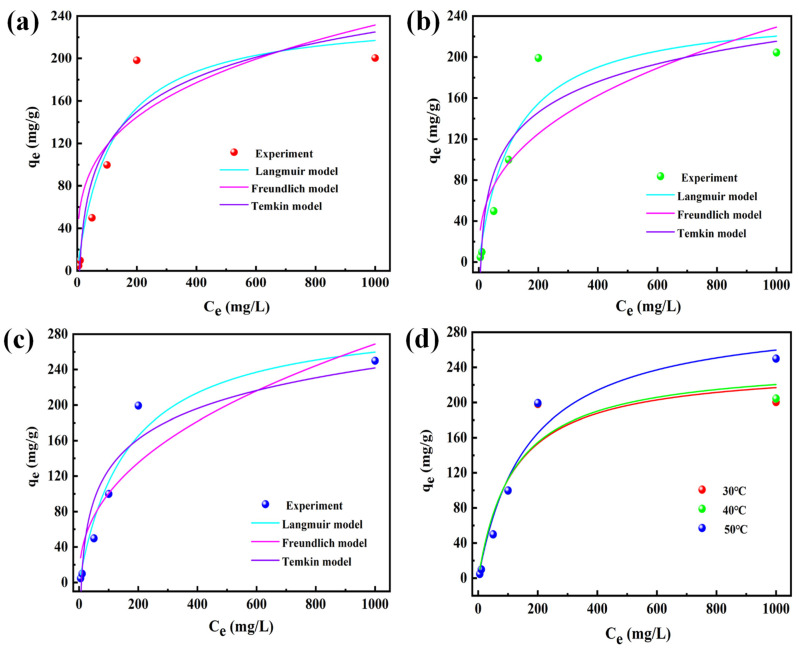
Fitting of Freundlich model, Langmuir model, and Temkin model: (**a**) under the condition of 30 °C; (**b**) under the condition of 40 °C; (**c**) under the condition of 50 °C; and (**d**) Langmuir model at different temperatures.

The aim of this study was to investigate the influence of different initial concentrations of fluoride ions and temperatures on the adsorption of fluoride ions. The adsorption thermodynamics were studied at 30 °C, 40 °C, and 50 °C by taking 5 mg/L, 10 mg/L, 50 mg/L, 100 mg/L, and 200 mg/L fluoride ion solutions. In this experiment, the Freundlich model, Langmuir model, Temkin model, and Dubinin–Radushkevich model were used for simulation. The fitting result of the Dubinin–Radushkevich model is displayed in Figure S3. The fitting results of three other models can be observed in Figure 3a–d. The parameters obtained through fitting are illustrated in Table S2. It could be seen from the fitting results that the adsorption thermodynamics of the adsorbed material for fluoride ions was more consistent with the Langmuir model. The phase-relationship values at the three temperatures of the Langmuir model (R^2^ = 0.901, 0.905, 0.953) were higher than those of the Freundlich model (R^2^ = 0.834, 0.741, 0.846) and the Temkin model (R^2^ = 0.886, 0.848, 0.885). This indicated that the removal process of fluoride ions by ternary composite metal oxides was mainly monolayer adsorption [43]. There might be some specific chemical functional groups on the surfaces of the adsorbed materials, which enabled fluoride ions to be adsorbed on the surface in the form of a single molecular layer. It would be further verified by other characterizations.

The fitting curve of the Langmuir model at different temperatures is presented in Figure 3d. The adsorption capacity of fluoride ions was enhanced by increasing the initial fluoride ion concentrations, and the adsorption performance exhibited robust stability across a temperature range of 5–200 mg/L. At the temperatures of 30 °C and 40 °C, the adsorption capacity of the composite material for fluoride ion concentrations of 200 mg/L and 1000 mg/L was not much different. This indicated that the adsorption equilibrium of the adsorption material of 50 mg had been reached, and the effective active sites of adsorbents had been effectively contacted with fluoride ions. Under the condition of 50 °C, the adsorption capacity of fluoride ions at the initial concentration of 1000 mg/L was higher than that at 30 °C and 40 °C. It indicated that the adsorption capacity improved with the increase in temperature when the initial concentration was higher than 200 mg/L. The findings indicated that the adsorption of fluoride ions by the composite adsorbent was an endothermic process, potentially transforming the inert sites of the adsorbent into active sites, consequently enhancing the adsorption capacity [44].

To further investigate the influence of different kinds of anions, five kinds of anions solutions were created, each with a fluoride ion concentration of 100 mg/L and additional competing anions (NO_3_^−^, SO_4_^2−^, Cl^−^, Br^−^, PO_4_^3−^) at a concentration of 10 mmol/L. The fluoride-removal efficiency of nano-ternary composite oxide materials under the competition of different anions is presented in Figure S4. It indicated that NO_3_^−^, SO_4_^2−^, Cl^−^, and Br^−^ had little effect on the adsorption and removal of fluoride ions by the material. The removal rate of fluoride ions was kept above 99%. The presence of PO_4_^3−^ had a significant impact on the removal rate of fluoride ions, resulting in a substantial competitive effect. As a result, the removal rate of fluoride ions only reached 7.5%. This phenomenon occurred because the PO_4_^3−^ ion carried a higher charge, facilitating its binding with H^+^ ions in the solution, thereby influencing the solution’s pH and consequently impacting the active sites of the ternary compound oxides [45,46]. In addition, the presence of PO_4_^3−^ affected the HF species of fluoride, limiting the effective contact between the fluoride ions and the adsorption active site. It could lead to a reduction in the adsorption efficiency.

### 3.2. Adsorption Mechanism

#### 3.2.1. Adsorption Characterization

**Figure 4:**
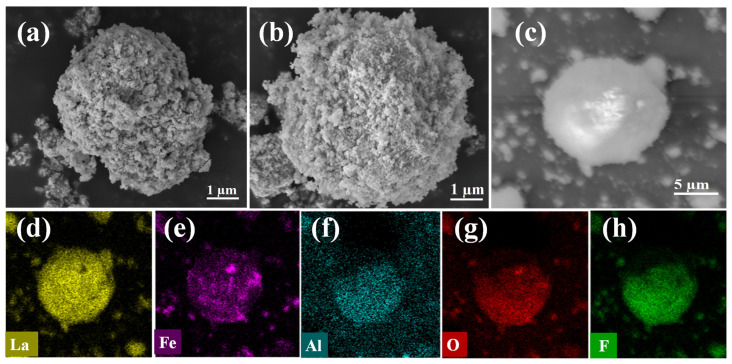
La-Fe-Al ternary composite oxides: (**a**) SEM image before adsorption of fluoride ions; (**b**) SEM image after adsorption of fluoride ions; (**c**) TEM image after adsorption of fluoride ions; and (**d**–**h**) EDS distributions of five elements, namely, La, Fe, Al, O, and F.

Compared with before and after adsorption (Figure 4a,b), the morphology of the material did not change significantly, indicating that the structure of the material was stable during the adsorption process. The La-Fe-Al ternary composite oxides showed the morphology of agglomeration particles before adsorption, and this agglomeration phenomenon was attributed to the mutual doping of the three metal oxides. It might be due to the attraction or interaction between nanoparticles, such as electrostatic force and van der Waals force interaction. The size of the particles after agglomeration was about 10 μm, accompanied by very small nano-round particles. Figure 4c shows the transmission electron microscopy (TEM) of La-Fe-Al ternary composite oxides after adsorption, and nanospheres could be observed. The element-mapping analysis was conducted based on the TEM image. From Figure 4d–g, it could be observed that Fe, La, Al, and O elements were uniformly dispersed in the spheroids, indicating that the three oxides were indeed attracted to each other and doped together. This explained the agglomeration. In addition, Figure 4h reveals that the F element was uniformly dispersed in the adsorbed material, indicating that the La-Fe-Al ternary composite oxide had a good adsorption effect on fluoride ions and was indeed a very ideal adsorbent, which was confirmed by the analysis of the removal rate of fluoride ions above 99% in Figure 1.

**Figure 5:**
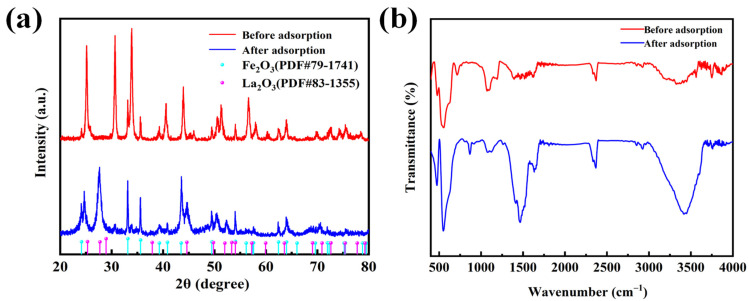
Before and after adsorption of fluoride ions by La-Fe-Al ternary composite oxides: (**a**) XRD pattern; (**b**) FT-IR diagram.

The XRD pattern of nano-ternary composite oxides before and after the adsorption of fluoride ions can be observed in Figure 5a. The material before adsorption was mainly composed of two phases, namely La_2_O_3_ (PDF#83-1355) and Fe_2_O_3_ (PDF#79-1741). The former was a tetragonal phase with a space group of P4/nmm (No. 129). The latter was a rhombohedral phase with a space group of R-3c (No. 167). Following the calcination process for the synthesis of the raw materials La_2_O_3_, FeCl_3_, and Al_2_(SO_4_)_3_, aluminum was not detected due to its formation of amorphous compounds. Upon adsorption of fluoride ions, the resulting adsorbent material consisted of Fe_2_O_3_ and La_2_O_3_, maintaining a stable structure before and after the adsorption process. The Fourier infrared spectroscopy (FT-IR) of nano-ternary composite oxides before and after the adsorption of fluoride ions was presented in Figure 5b. There was an obvious peak at the wavelength of 3429 cm^−1^, accompanied by adsorption peaks at 1470 cm^−1^ and 1098 cm^−1^, which exhibited a characteristic O-H stretch band. It indicated that the material contained hydroxy groups [47]. By comparing the infrared spectra before and after adsorption, it was found that the peak strength of the hydroxyl group before adsorption was weaker than that after adsorption, in the range of 3200–3600 cm^−1^. This occurred due to the increased presence of water-bonding bonds in the material after adsorption, as the material was not fully dried, resulting in a stronger peak intensity of the hydroxyl group after adsorption. When the wavelength was 452 cm^−1^, the adsorbed material had an extra peak, indicating that the binding bond between the metal and fluoride ions was formed after the adsorption of fluoride ions by the material. Thus, the adsorption peak appeared. In addition, the position of the adsorption peak did not change before and after adsorption. That was the functional group which did not change, indicating that its performance was stable.

The TG diagram of nano-ternary composite oxides before and after adsorption is illustrated in Figure S5. The material exhibited minimal weight loss as the temperature increased, suggesting its stability prior to adsorption. In contrast, the adsorbed material showed more pronounced weight loss, with rising temperatures compared to its pre-adsorption state. This observation indicated that the H-F bond formed through adsorption, and the bonding between the metal and fluoride ions had decomposition. During this interval, the decrease in weight could be attributed to water evaporation, reducing from 100% to around 97% within the temperature range of 30 °C to 100 °C. Furthermore, there were three clearly defined weight-loss stages observed from 100 °C to 800 °C. Within the range of 100 °C to 200 °C, the weight percentage dropped to about 96% as the temperature rose. Between 300 °C and 600 °C, the weight percentage decreased from 94% to 88%. Within the range of 700 °C to 800 °C, the weight percentage dropped from 87% to 85%. The three weight loss gradients were the losses of the bonding bonds between the Al, Fe, and La metals, and fluoride ions.

#### 3.2.2. Changes in X-ray Photoelectron Spectroscopy

**Figure 6:**
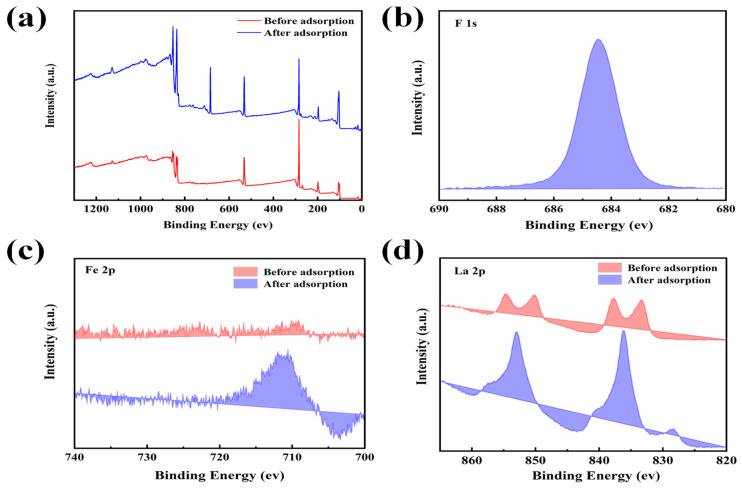
La-Fe-Al ternary composite oxides: (**a**) full spectrum analysis of XPS before and after adsorption; (**b**) high-resolution spectra of F 1s after adsorption; (**c**) high-resolution spectra of Fe 2p before and after adsorption; (**d**) high-resolution spectra of La 2p before and after adsorption.

The detailed surface composition and chemical state of the adsorbent during adsorption could be elucidated through XPS characterization. The XPS full-spectrum analysis of the La-Fe-Al ternary composite oxides before and after adsorption is illustrated in Figure 6a. After adsorption, there was an obvious peak at 685 eV, where the peak was F 1s, indicating that the material adsorbed fluoride ions to form a new binding bond. The F 1s spectrum after the adsorption of fluoride ions by the material can be observed from Figure 6b, and the center of the peak was about 685 eV [48]. The La-Fe-Al ternary composite oxides exhibited a potent adsorption capacity for fluoride ions, effectively immobilizing them on the surface and within the microporous structure of the material.

The high-resolution map of Fe 2p is illustrated in Figure 6c. Before the adsorption, the material had an obvious bimodal shape. However, the bimodal change was very irregular after the adsorption of fluoride ions, which indicated that Fe played a very important role in the adsorption process. It could promote the binding of fluoride ions and iron sites in the solution. The high-resolution spectrum of La 2p is presented in Figure 6d. The two adjacent peaks were combined into one because La and F were combined to form the La-F bond after adsorption. The high-resolution spectrum of Al 2p can be seen in Figure S6. The shift towards lower binding energy indicated that Al also played an important role in the adsorption process.

### 3.3. Density Functional Theory Calculation

**Figure 7:**
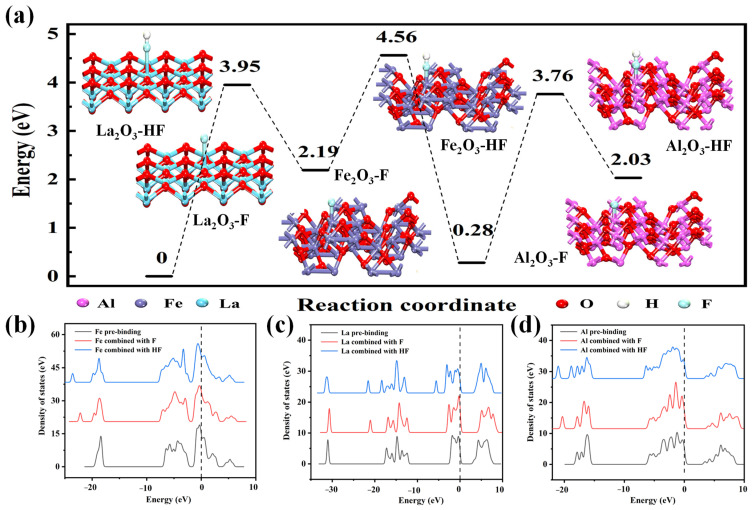
(**a**) The adsorption relationship between La_2_O_3_, Fe_2_O_3_, Al_2_O_3_, and different fluoride ion forms; (**b**) the change of total state density after Fe was combined with fluoride in different ionic forms; (**c**) the change of total state density after the combination of La and fluoride with different ionic forms; (**d**) the change of total state density of Al combined with fluoride in different ionic forms.

Through DFT calculation, the main reactions in the adsorption process could be revealed, and the mechanism could be interpreted better. As shown in Figure 7a, the binding energy of La_2_O_3_, Fe_2_O_3_, and Al_2_O_3_ for HF adsorption is 3.95 eV, 4.56 eV, and 3.76 eV, respectively. Due to the facile formation of HF in acidic fluoride ion solutions, it was evident that La and Fe served as the primary adsorption sites on the synthesized nano-ternary composite during the adsorption of HF. In addition, the adsorption energies of the three oxides for F^−^ were 2.19 eV, 0.28 eV, and 2.03 eV, respectively. It indicated that La and Al were more important adsorption sites in the adsorption process of F^−^ [49]. In general, the adsorptive ability of HF is Fe_2_O_3_ > La_2_O_3_ > Al_2_O_3_; the adsorptive ability of F^−^ is La_2_O_3_ > Al_2_O_3_ > Fe_2_O_3_.

**Figure 8:**
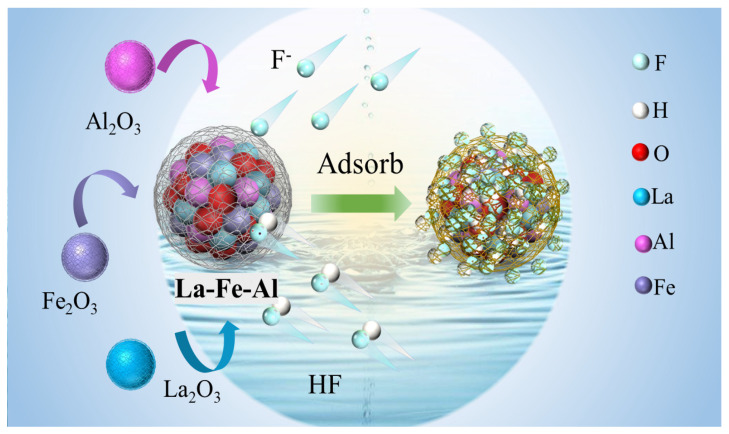
The adsorption mechanism of La-Fe-Al ternary composite oxides.

The change of the total state density after Fe was combined with fluoride as different ion species is illustrated in Figure 7b. It could be found that, after Fe and F^−^ were combined, there was no change in the band position and state density near the Fermi level. The combination of Fe and HF resulted in a leftward shift in the position near the Fermi level. This observation aligns with the findings presented in Figure 7a, indicating the significant role of Fe in the adsorption of HF. The change of total state density after La was combined with fluoride as different ion species in Figure 7c. It could be observed that, after La adsorbed F^−^ or HF, the band position shifted to the left. A new peak pattern appeared at the Fermi interface, which indicated that fluoride ions were effectively adsorbed by La. It formed the La-F or La-HF structure. The change of total state density after Al was combined with fluoride ions as different species is presented in Figure 7d. The peak value at the Fermi level changed greatly before and after adsorption, which indicated that Al played an important role in the adsorption of fluoride ions, even though the proportion of Al in the ternary complex products was very small. In addition, the state-density diagram of each orbital of La, Fe, and Al is illustrated in Figures S7–S14. It could be found that the adsorption of fluoride ions at the La and Fe sites was mainly caused by the change of electrons in the p and d orbitals. However, the change of electrons in the p orbitals at the Al site was mainly caused by the change of electrons in the p orbitals, resulting in the recombination of orbitals and promoting the adsorption reaction. In summary, the attraction generated by the electron changes in the outer layers of La, Fe, and Al determined the removal performance of fluoride ions. Therefore, the adsorption mechanism of binding F^−^ and HF with La-Fe-Al ternary composite oxides in the adsorption phase diagram is revealed in Figure 8.

## 4. Conclusions

In this study, based on the treatment of fluoride-containing wastewater, the effect of the doping ratio of La_2_O_3_, Fe_2_O_3_, and Al_2_O_3_ on the fluoride-removal performance was discussed by constructing a phase diagram. It was found that the optimal mass ratio of nano-binary composite oxides to fluoride ions was Fe_2_O_3_:La_2_O_3_ = 1:2, and the removal rate of fluoride ions could reach 99.56%. The optimal mass ratio of nano-ternary composite oxides to fluoride ions was Al_2_O_3_:(Fe_2_O_3_:La_2_O_3_ = 1:2) = 1:100, and the removal rate of fluoride ions could reach 99.78%. The optimum pH was three, and the adsorption rate was 99.2%. The La-Fe-Al ternary composite oxides synthesized using the phase-diagram method exhibited excellent stability, with no changes in morphology or structure observed before and after adsorption. In addition, the composite product had three important adsorption sites: La, Fe, and Al. The adsorptive ability of HF was Fe_2_O_3_ > La_2_O_3_ > Al_2_O_3_, and the adsorptive ability of F^−^ La_2_O_3_ > Al_2_O_3_ > Fe_2_O_3_. In summary, the La-Fe-Al ternary composite oxides synthesized using the ternary phase-diagram method in this study exhibited a highly significant adsorption effect on fluoride ions. This method provides a certain reference significance for the synthesis of other superior adsorbents.

## Data Availability

Data will be made available on request.

## Supplementary Materials

The following supporting information can be downloaded at https://www.mdpi.com/article/10.3390/nano14070619/s1, Figure S1. Fluoride ions adsorption kinetics curves of La-Fe-Al ternary composite oxides; Figure S2. Fitting curve of Elovich kinetic model; Figure S3. Fitting curve of Dubinin-Radushkevich model; Figure S4. Removal efficiency of La-Fe-Al ternary composite oxides under competition of different anions; Figure S5. The TG diagram before and after adsorption of La-Fe-Al ternary composite oxides; Figure S6. High-resolution map of Al 2p; Figure S7. The density changes of s orbital states of La before and after adsorption; Figure S8. The density changes of p orbital states of La before and after adsorption; Figure S9. The density changes of d orbital states of La before and after adsorption; Figure S10. The density changes of s orbital states of Fe before and after adsorption; Figure S11. The density changes of p orbital states of Fe before and after adsorption; Figure S12. The density changes of d orbital states of Fe before and after adsorption; Figure S13. The density changes of s orbital states of Al before and after adsorption; Figure S14. The density changes of p orbital states of Al before and after adsorption; Table S1: The Kinetic models of La-Fe-Al ternary composite oxides; Table S2. The thermodynamic model of La-Fe-Al ternary composite oxides at different temperatures.
