# Supplementary material for: The Constructing of the Oxide Phase Diagram for Fluoride Adsorption on La-Fe-Al: A Collaborative Study of Density Functional Calculation and Experimentation
Source: Nanomaterials (Basel). 2024 Apr 1;14(7):619. doi: 10.3390/nano14070619 (PMC11013458; doi:10.3390/nano14070619)
Supplement: Supplementary file 1 [file nanomaterials-14-00619-s001.zip › nanomaterials-2930972-supplementary.pdf]

Supplementary Material

The Constructing of the Oxide Phase Diagram for Fluoride Adsorption on La-Fe-Al: A Collaborative Study of Density Functional Calculation and Experimentation

Shaojian Xie [1,†], Yao Xiao [1,†], Lei Huang [1,*], Jiaxin Li [1], Jia Yan [1], Qian Li [1], Meng Li [1] and Hongguo Zhang [1,2,*]

[1] School of Environmental Science and Engineering, Guangzhou University, Guangzhou Higher Education Mega Center, Guangzhou 510006, China
[2] Guangzhou University-Linköping University Research Center on Urban Sustainable Development, Guangzhou University, Guangzhou 510006, China
* Correspondence: huanglei@gzhu.edu.cn (L.H.); hgzhang@gzhu.edu.cn (H.Z.)
† These authors contributed equally to this work.

Supplementary Figures:

Figure S1. Fluoride ions adsorption kinetics curves of La-Fe-Al ternary composite oxides.

Figure S2. Fitting curve of Elovich kinetic model.

Figure S3. Fitting curve of Dubinin-Radushkevich model.

Figure S4. Removal efficiency of La-Fe-Al ternary composite oxides under competition of different anions.

Figure S5. The TG diagram before and after adsorption of La-Fe-Al ternary composite oxides.

Figure S6. High-resolution map of Al 2p.

Figure S7. The density changes of s orbital states of La before and after adsorption.

Figure S8. The density changes of p orbital states of La before and after adsorption.

Figure S9. The density changes of d orbital states of La before and after adsorption.

Figure S10. The density changes of s orbital states of Fe before and after adsorption.

Figure S11. The density changes of p orbital states of Fe before and after adsorption.

Figure S12. The density changes of d orbital states of Fe before and after adsorption.

Figure S13. The density changes of s orbital states of Al before and after adsorption.

Figure S14. The density changes of p orbital states of Al before and after adsorption.

Supplementary Tables:

Table S1. The Kinetic models of La-Fe-Al ternary composite oxides.

Table S2. The thermodynamic model of La-Fe-Al ternary composite oxides at different temperatures.

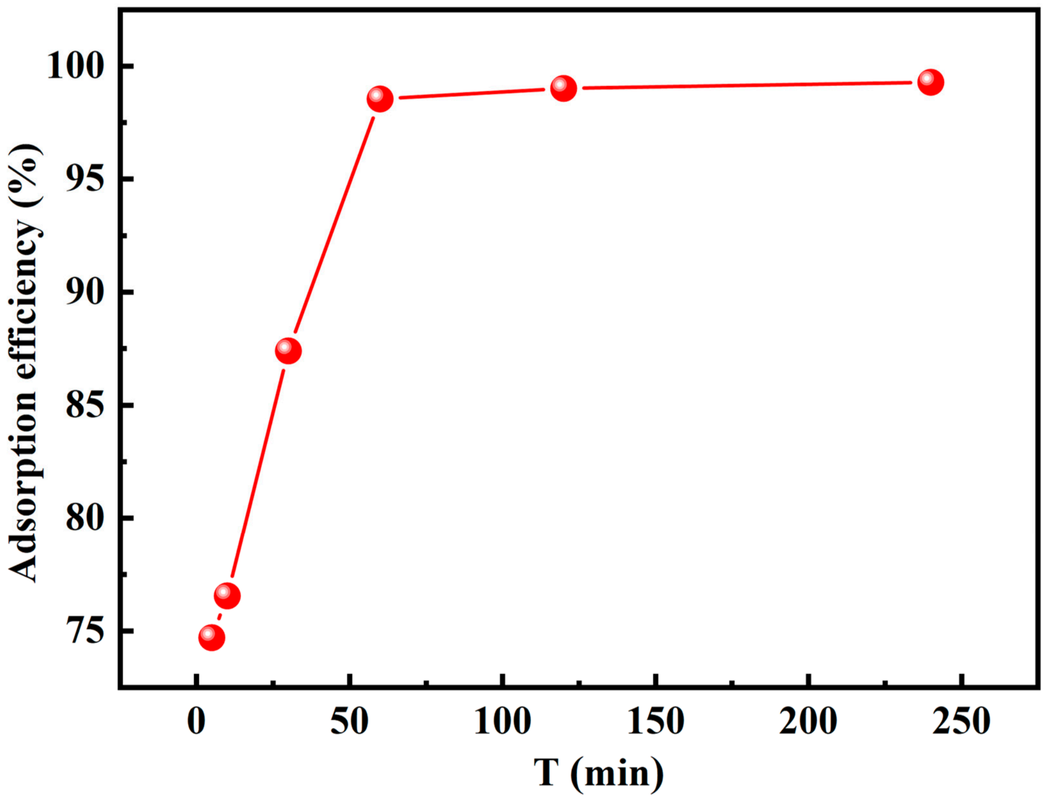

Figure S1. Fluoride ions adsorption kinetics curves of La-Fe-Al ternary composite oxides.

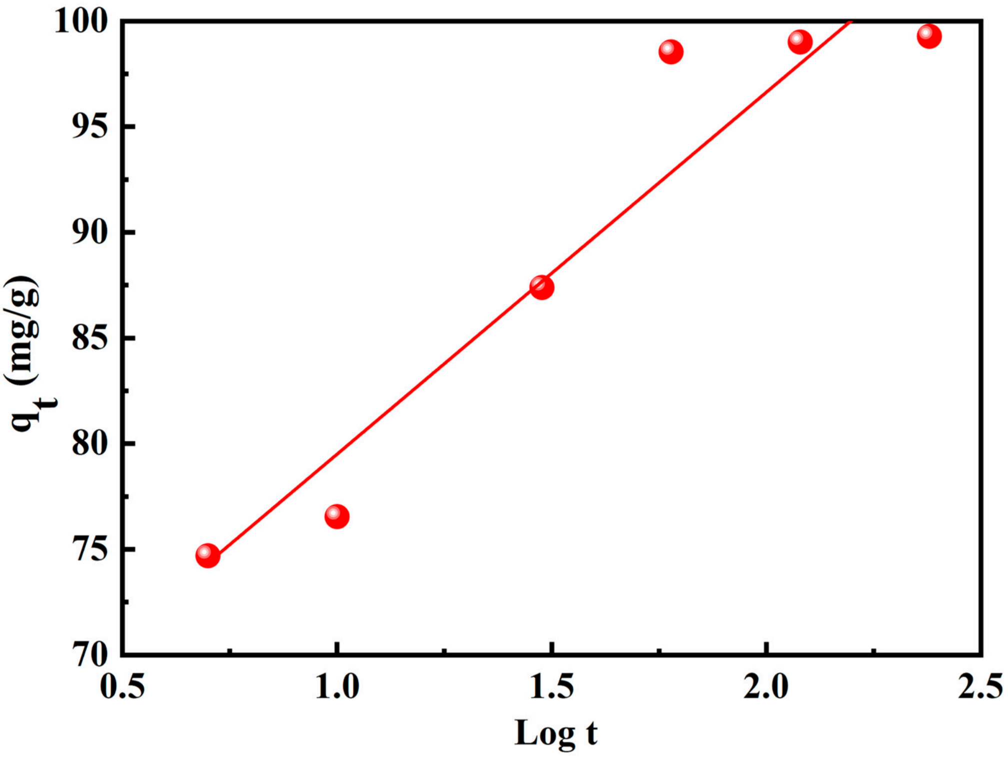

Figure S2. Fitting curve of Elovich kinetic model.

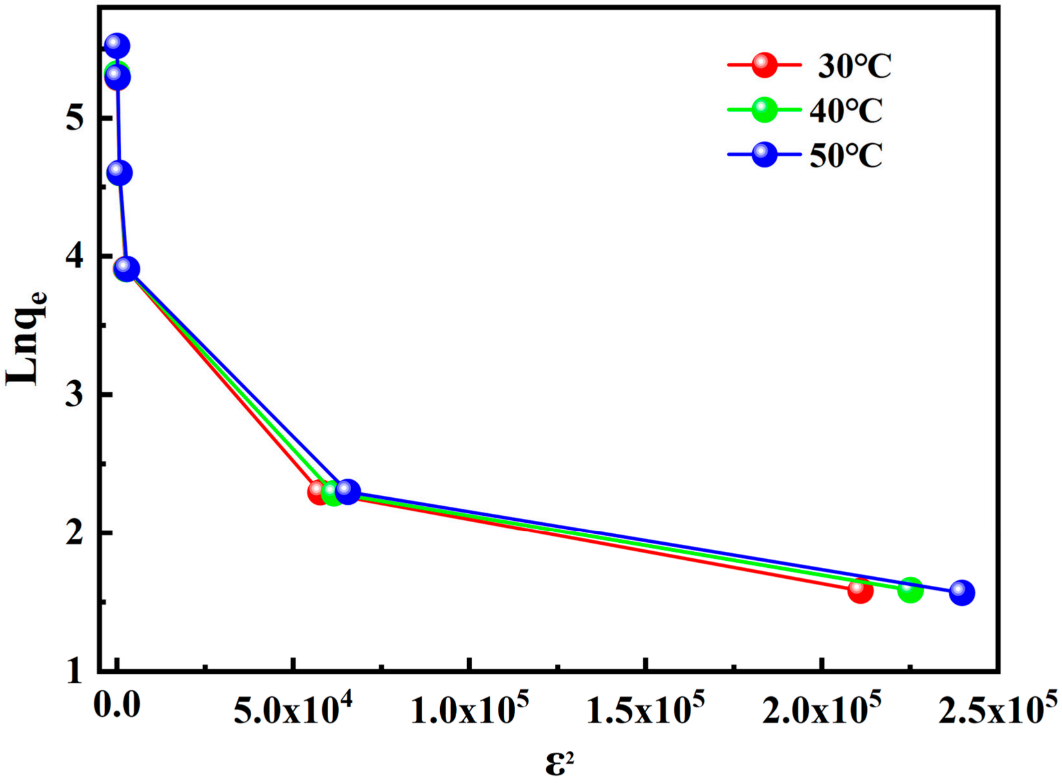

Figure S3. Fitting curve of Dubinin-Radushkevich model.

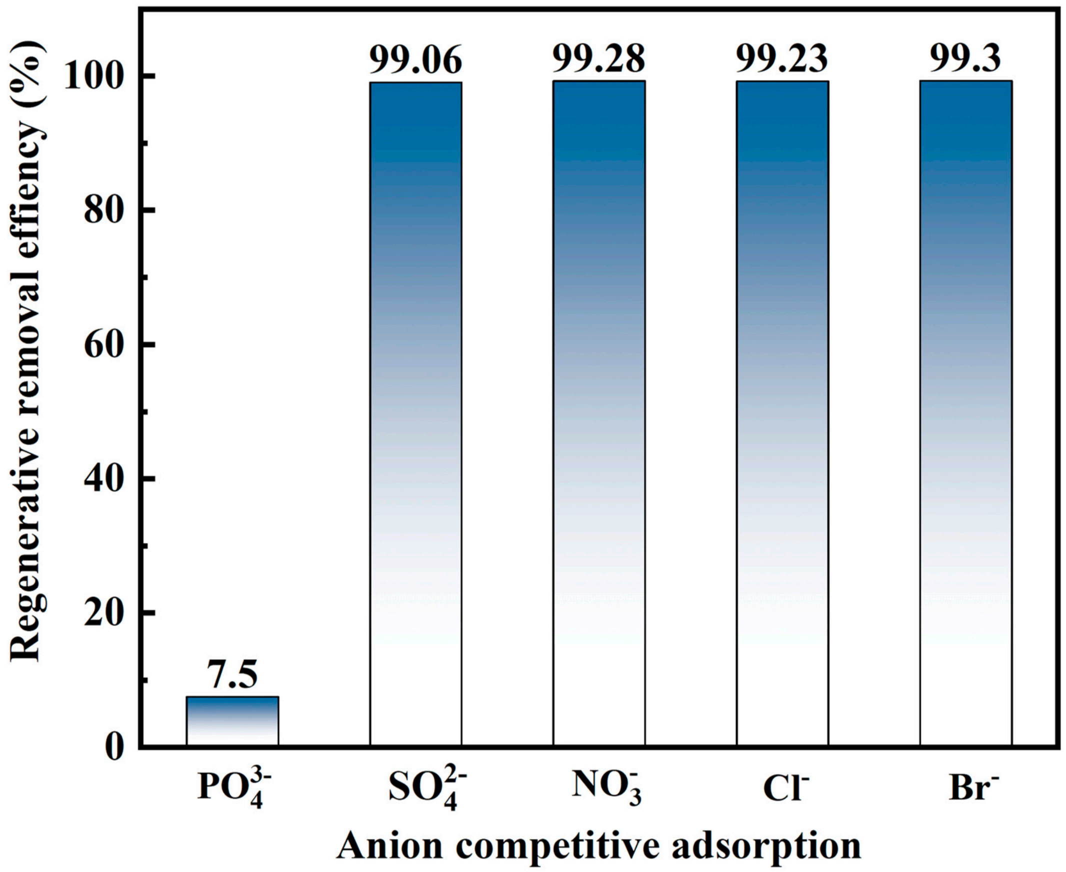

Figure S4. Removal efficiency of La-Fe-Al ternary composite oxides under competition of different anions.

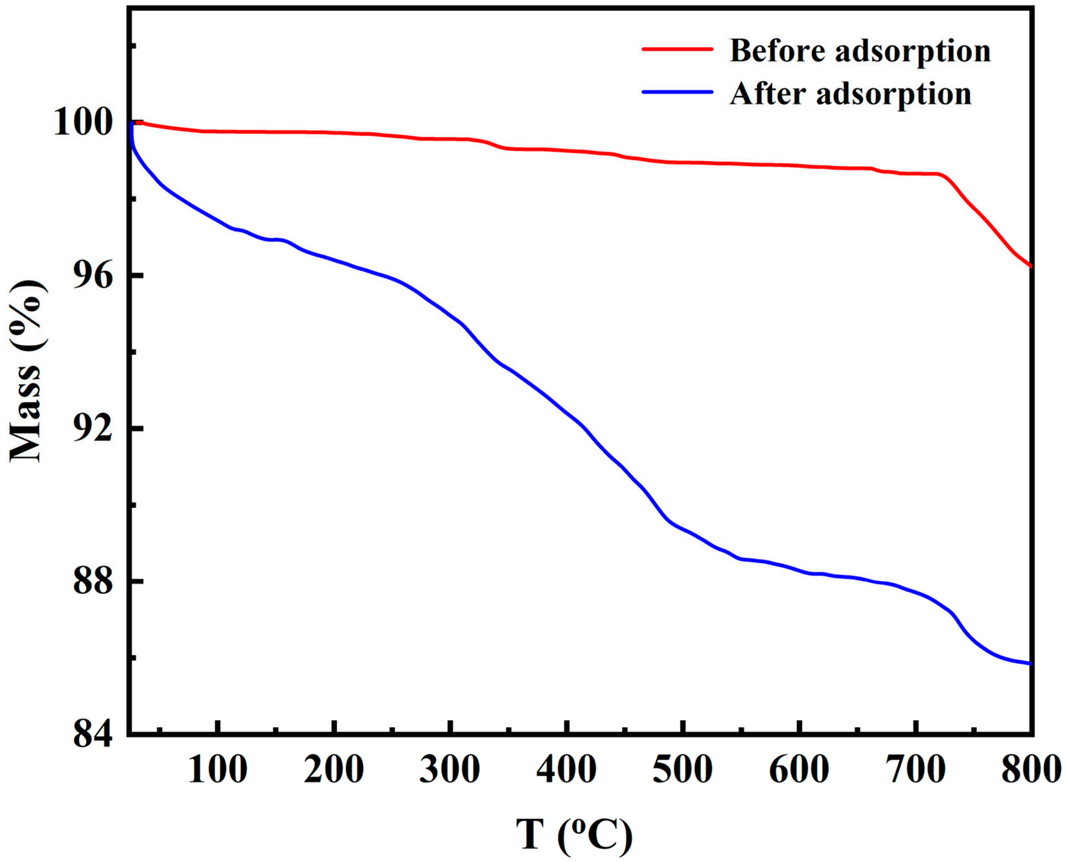

Figure S5. The TG diagram before and after adsorption of La-Fe-Al ternary composite oxides.

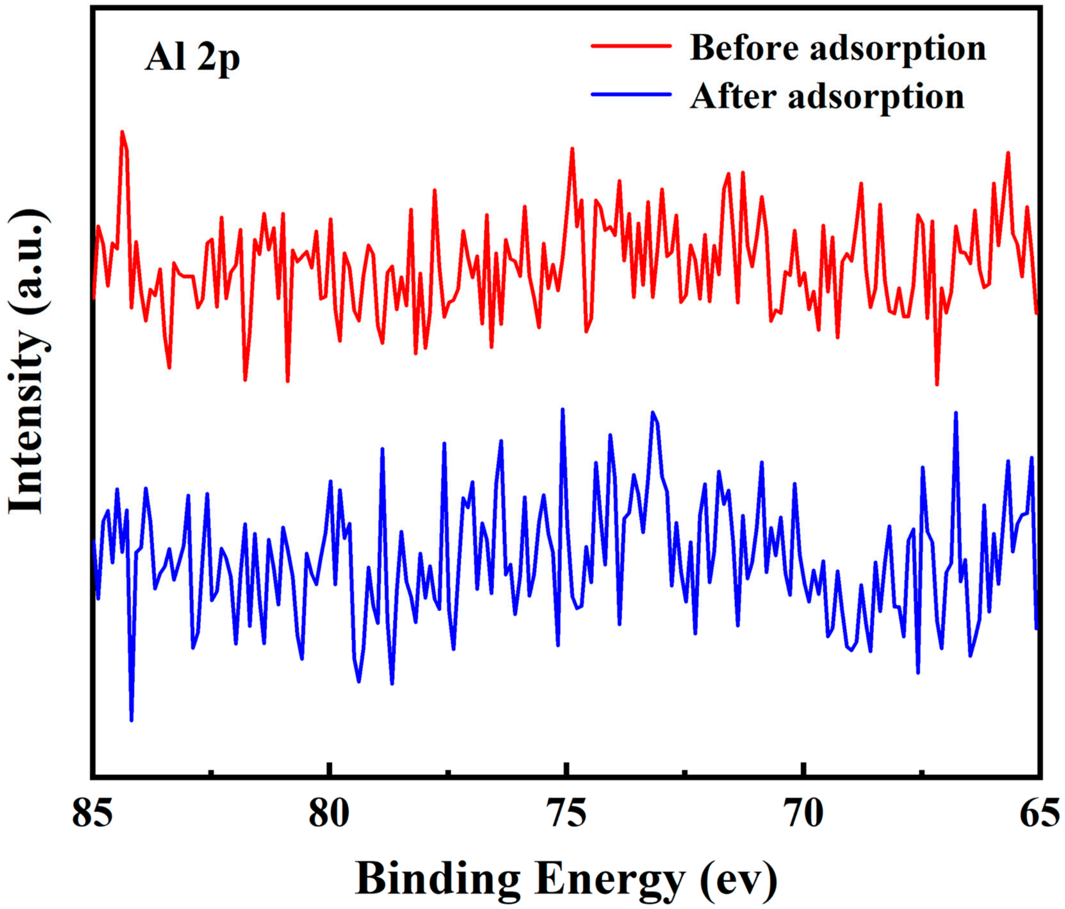

Figure S6. High-resolution map of Al 2p.

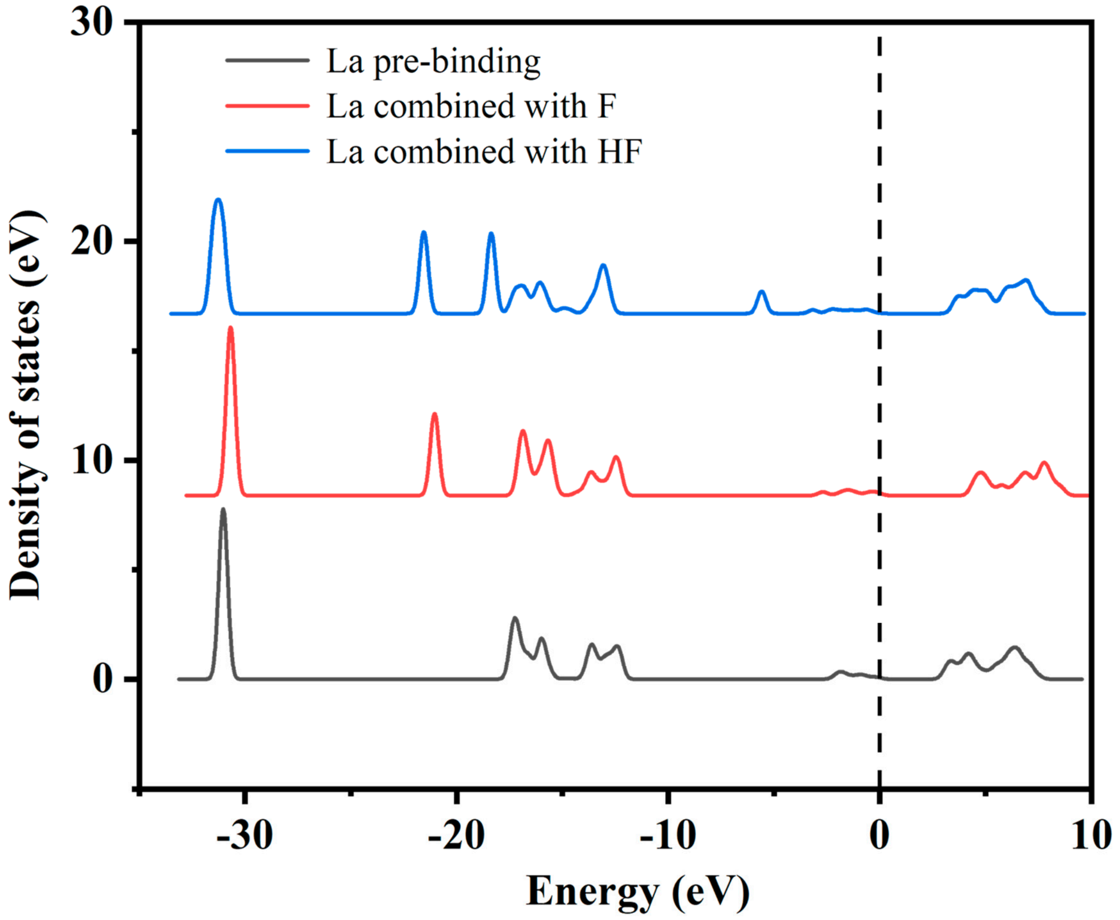

Figure S7. The density changes of s orbital states of La before and after adsorption.

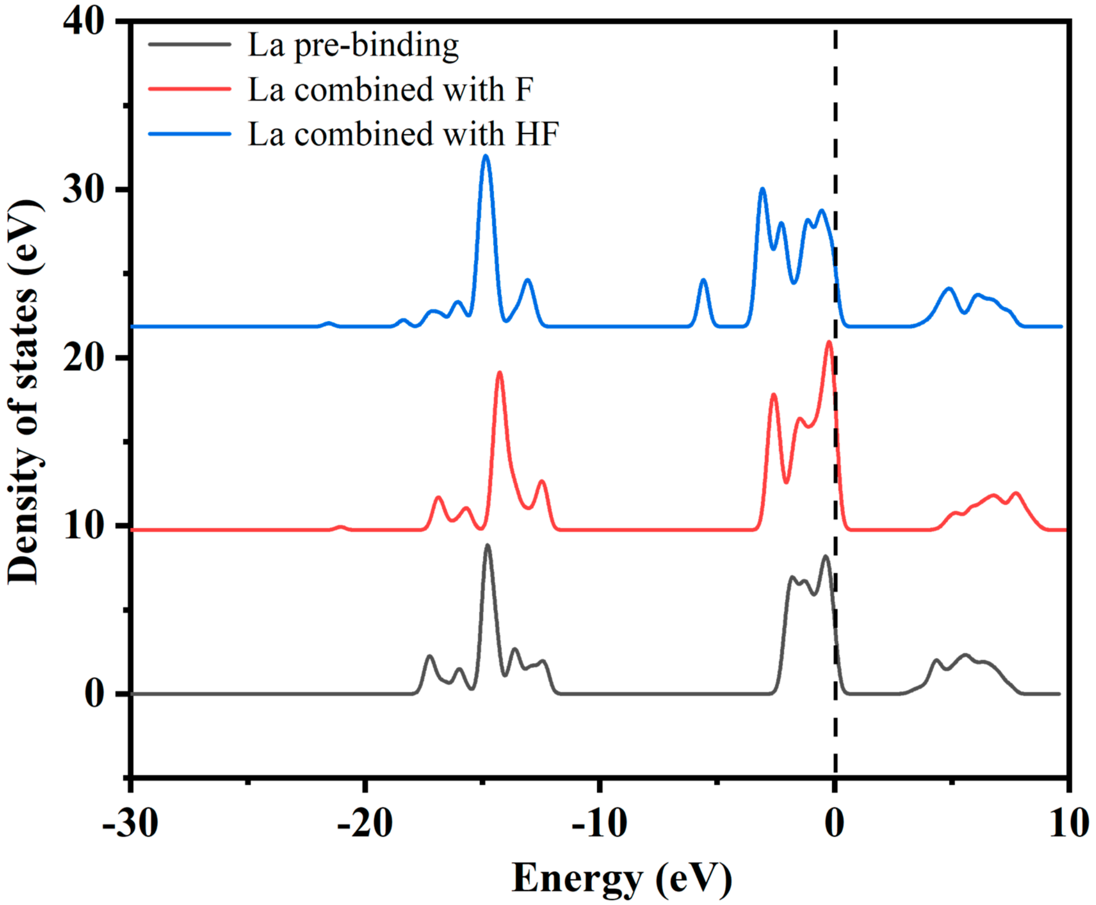

Figure S8. The density changes of p orbital states of La before and after adsorption.

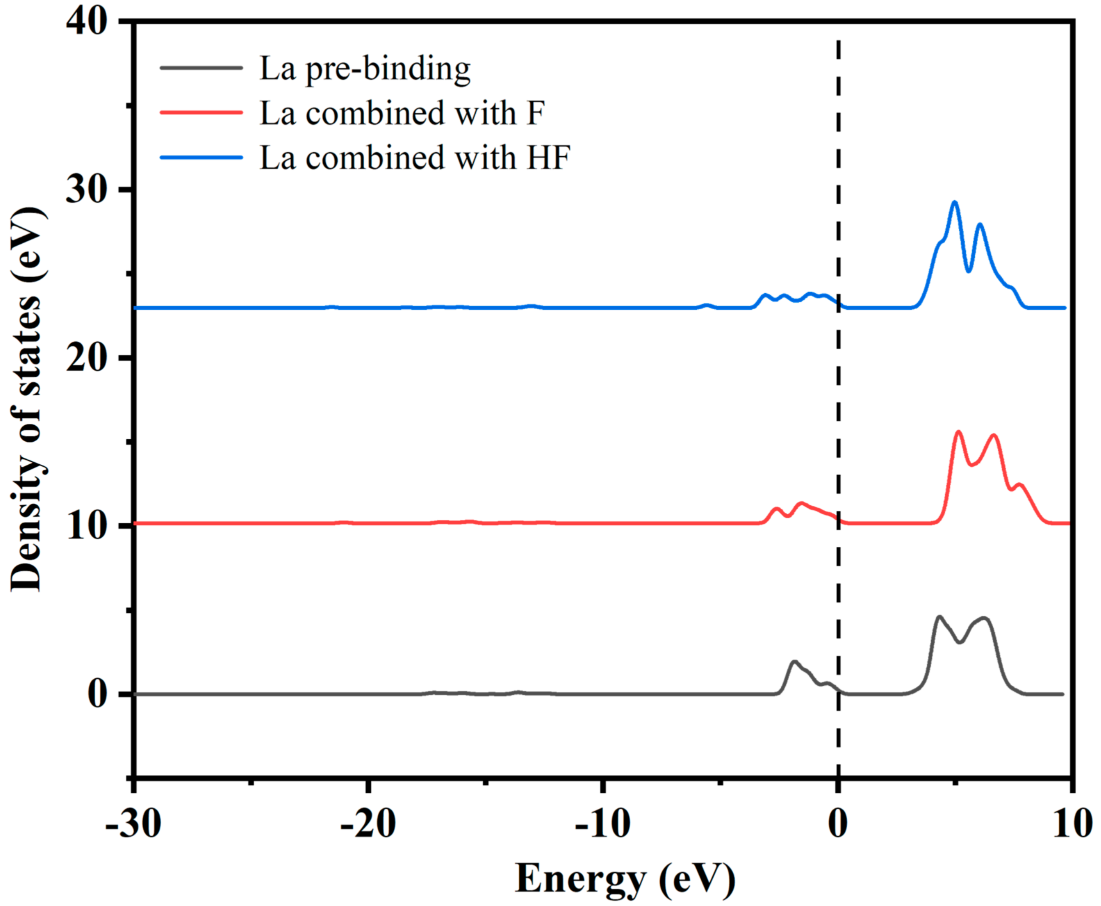

Figure S9. The density changes of d orbital states of La before and after adsorption.

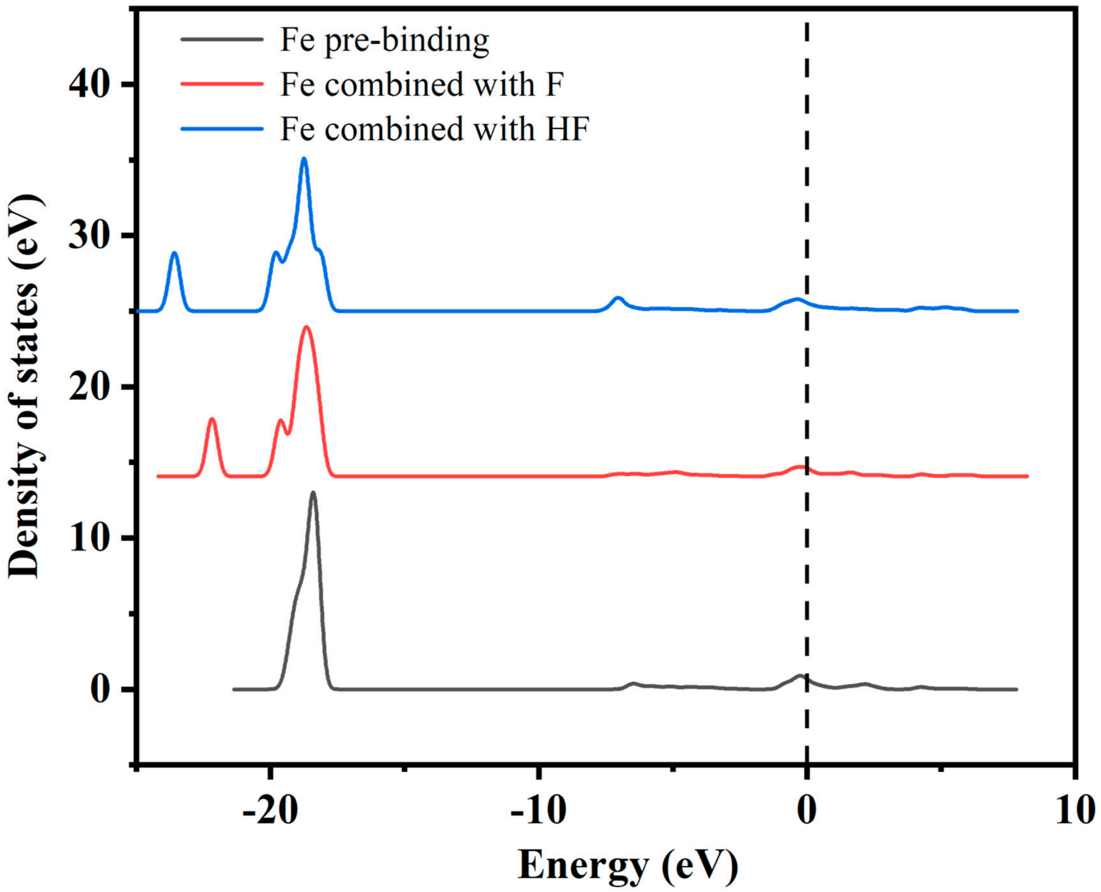

Figure S10. The density changes of s orbital states of Fe before and after adsorption.

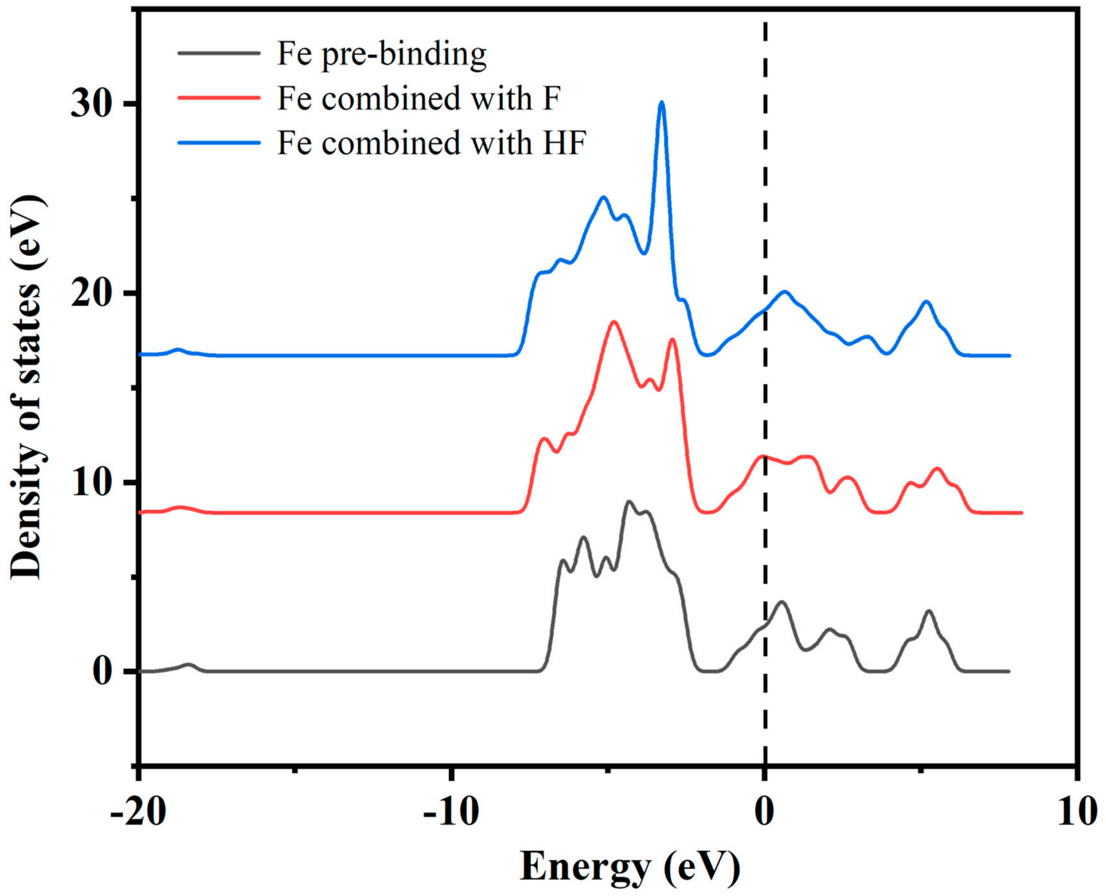

Figure S11. The density changes of p orbital states of Fe before and after adsorption.

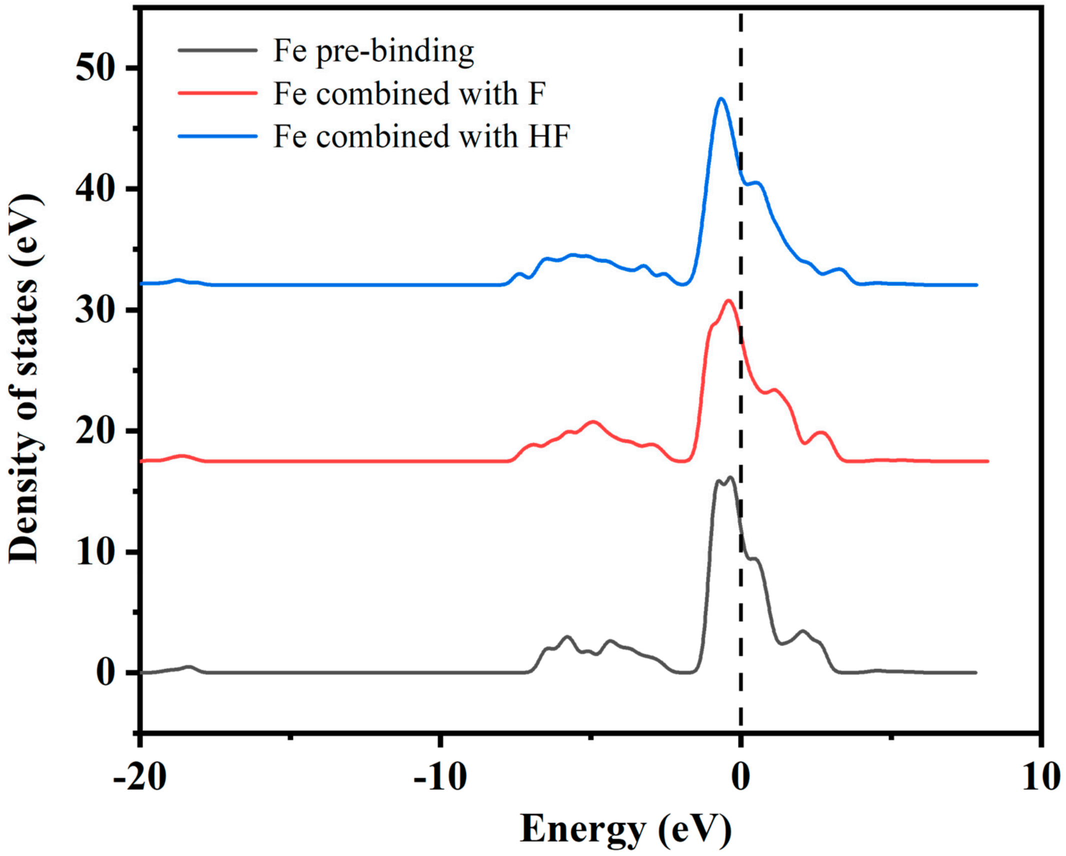

Figure S12. The density changes of d orbital states of Fe before and after adsorption.

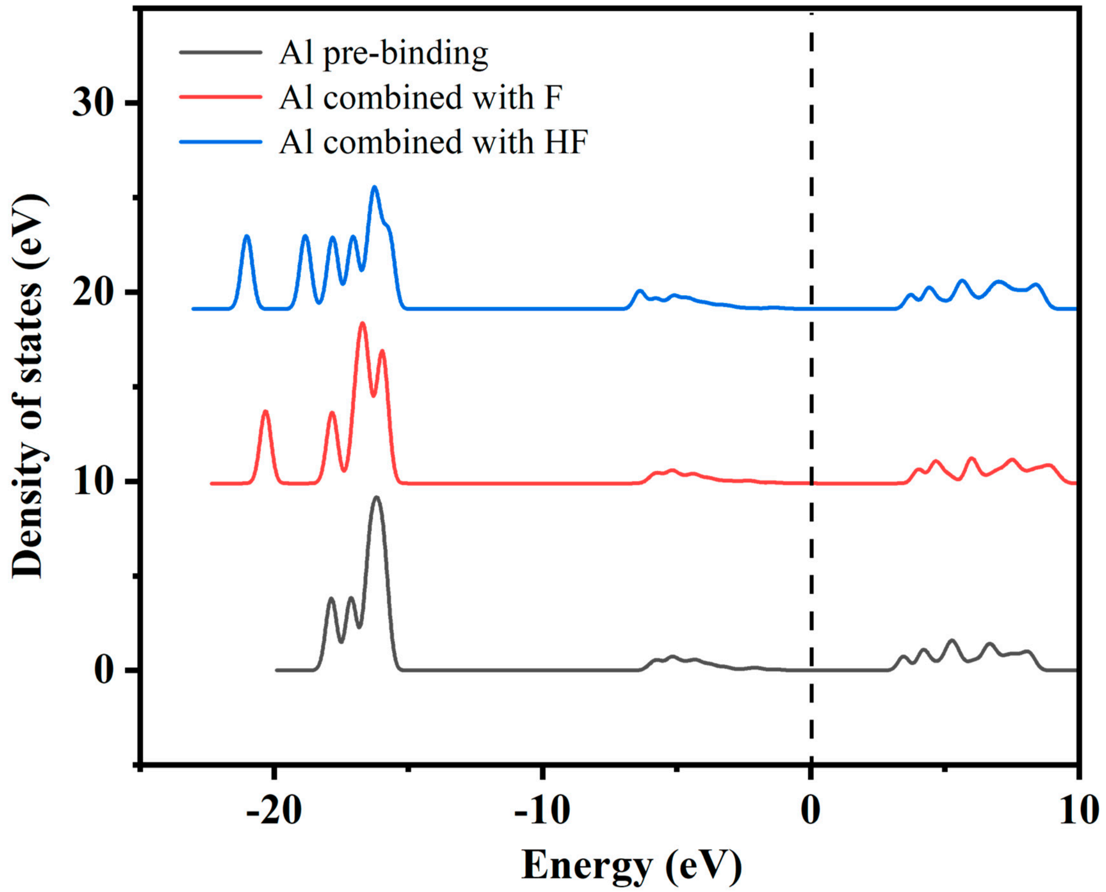

Figure S13. The density changes of s orbital states of Al before and after adsorption.

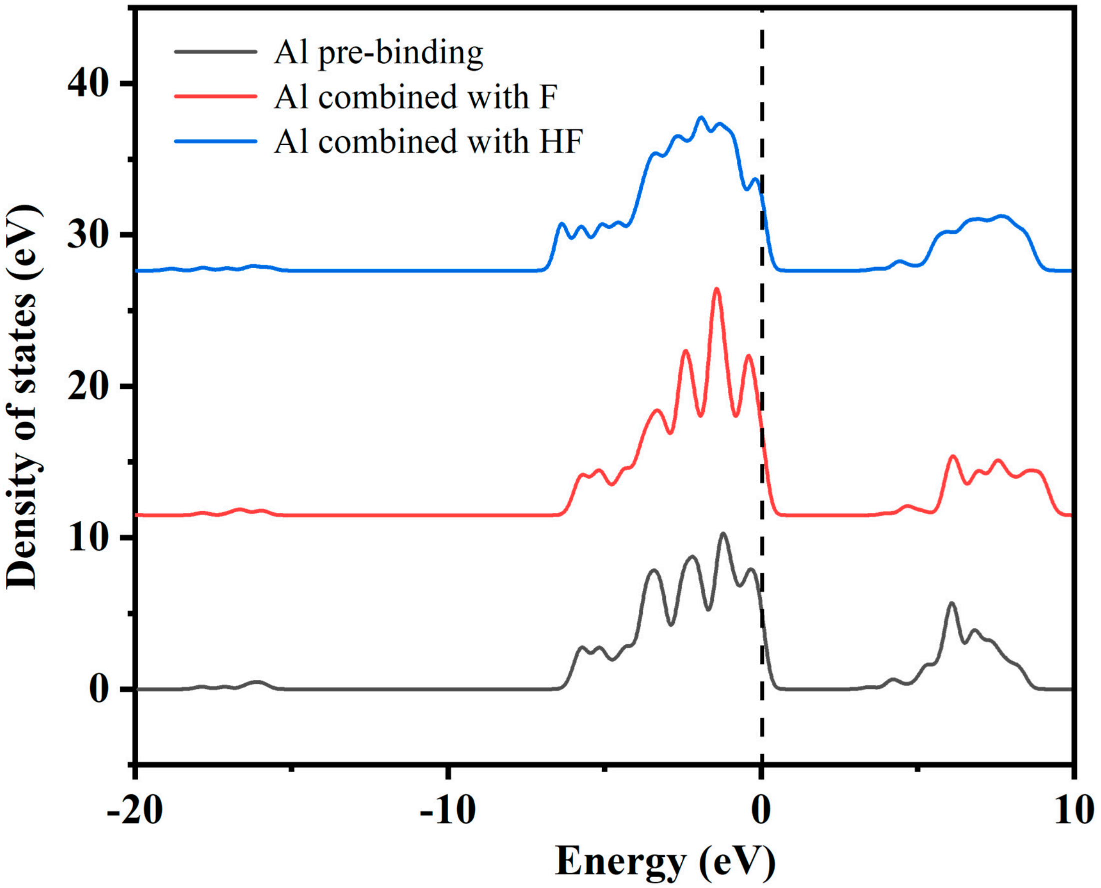

Figure S14. The density changes of p orbital states of Al before and after adsorption.

Table 1. The Kinetic models of La-Fe-Al ternary composite oxides.

**Pseudo-first-order model**				
C_o(mg/L)	Function	k_1(min^{-1})		R^2
100	$\log(q_e - q_t) = \log q_e - \frac{k_1 t}{2.303}$	0.042		0.876
**Pseudo-second-order model**				
C_o(mg/L)	Function	k_2($g \cdot mg^{-1} \cdot min^{-1}$)	q_e(cal)(mg/g)	R^2
100	$\frac{t}{q_t} = \frac{1}{k_2 q_e^2} + \frac{t}{q_e}$	0.0039	100.50	0.999
**Elovich Equation**				
C_o(mg/L)	Function	A	B	R^2
100	q_t=A+2.303Blogt	62.383	7.436	0.891
**Intraparticle Diffusion Kinetic Equation**				
C_o(mg/L)	Function	k_{id}($mg \cdot g^{-1} \cdot min^{-0.5}$)	C	R^2
100	$q_t = k_{id} \cdot t^{\frac{1}{2}} + C$	1.990	74.304	0.700
**External Diffusion Kinetic Equation**				
C_o(mg/L)	Function	k_p (min^{-1})		R^2
100	$\text{In}\frac{C_t}{C_0} = -K_p t$	0.016		0.639

Table 2. The thermodynamic model of La-Fe-Al ternary composite oxides at different temperatures.

Temperature(℃)	Langmuir model			Freundlich model			Temkin model		
	$q_e = \frac{q_{max} b C_e}{1 + b C_e}$			$q_e = K_F C_e^{1/n}$			$q_e = \frac{RT}{b_T} \ln(K_T T C_e)$		
	R^2	q_m (mg·g^{-1})	b (L·mg^{-1})	R^2	K_F (mg$^{1-(1/n)}$L$^{1/n}$g^{-1})	n	R^2	K_T	b_T
30	0.901	241.96	0.0087	0.834	30.83	3.43	0.886	0.00041	53.95
40	0.905	246.64	0.0084	0.741	17.12	2.66	0.848	0.00047	60.38
50	0.954	302.99	0.0060	0.846	14.07	2.34	0.885	0.00040	53.91
